# Dementia-linked amyloidosis is associated with brain protein deamidation as revealed by proteomic profiling of human brain tissues

**DOI:** 10.1186/s13041-016-0200-z

**Published:** 2016-02-19

**Authors:** Sunil S. Adav, Xavier Gallart-Palau, Kok Hian Tan, Sai Kiang Lim, James P. Tam, Siu Kwan Sze

**Affiliations:** School of Biological Sciences, Division of Structural Biology and Biochemistry, Nanyang Technological University, 60 Nanyang Drive, Singapore, 637551 Singapore; Department of Maternal Fetal Medicine, KK Women’s and Children’s Hospital, 100 Bukit Timah Road, 229899 Singapore, Singapore; Institute of Medical Biology, A*STAR, 8A Biomedical Grove, 138648 Singapore, Singapore

**Keywords:** Neurodegenerative disease, Amyloids, Deamidation, Brain Proteome, Protein S100A9, Mitochondrial creatine kinase

## Abstract

**Background:**

Aggregation of malformed proteins is a key feature of many neurodegenerative diseases, but the mechanisms that drive proteinopathy in the brain are poorly understood. We aimed to characterize aggregated proteins in human brain tissues affected by dementia.

**Results:**

To characterize amyloidal plaque purified from post-mortem brain tissue of dementia patient, we applied ultracentrifugation-electrostatic repulsion hydrophilic interaction chromatography (UC-ERLIC) coupled mass spectrometry-based proteomics technologies. Proteomics profiling of both soluble and aggregated amyloidal plaque demonstrated significant enrichment and deamidation of S100A9, ferritin, hemoglobin subunits, creatine kinase and collagen protein among the aggregated brain proteins. Amyloidal plaques were enriched in the deamidated variant of protein S100A9, and structural analysis indicated that both the low- and high-affinity calcium binding motifs of S100A9 were deamidated exclusively in the aggregated fraction, suggesting altered charge state and function of this protein in brain tissues affected by dementia. The multiple deamidated residues of S100A9 predicts introduction of negative charge that alter Ca^++^ binding, suggesting increased capacity to form pathological aggregates in the brain.

**Conclusion:**

UC-coupled proteomics revealed that brain amyloidal plaques are enriched in deamidated proteins, and suggested that altered charge state and calcium-binding capacity of S100A9 may enhance protein aggregation and promote neurodegeneration in the human brain.

**Electronic supplementary material:**

The online version of this article (doi:10.1186/s13041-016-0200-z) contains supplementary material, which is available to authorized users.

## Background

Proteinopathy is caused by the aggregation of malformed proteins (amyloids) to form insoluble plaques in the brain, leading to neurodegenerative pathology in disorders including Alzheimer’s disease (AD), Parkinson’s disease (PD), and Huntington’s disease (HD) [1, 2]. Accumulation of abnormally-folded *tau* protein and amyloid plaque formation are typical features of AD [2, 3], while accumulation of α-synuclein protein triggers death of dopamine-generating cells in the substantia nigra of PD patients [4], and intracellular aggregates of Huntingtin (Htt) protein causes HD [5]. Accordingly, it is now widely accepted that changes in brain protein function and aggregation are key features of multiple neurodegenerative diseases, but the specific protein modifications that first initiate and promote plaque formation are poorly defined [6]. In order to pave the way for effective new therapies that can protect against proteinopathy and disrupt neurodegeneration in human patients, it will first be necessary to identify the key disease-associated changes in protein relative abundance, solubility, and neurotoxicity in human brain tissues affected by dementia.

Accurate identification and quantitation of amyloid proteins in brain tissue extracts is technically challenging due to the ability of these molecules to self-associate and form multiple variants, each with distinct properties and variable solubility [7]. However, overcoming these technical difficulties will be vital to understanding the biological basis of synapse loss and human cognitive impairment, which has already been linked with the accumulation of soluble neurotoxic amyloids in the brain [8]. Accordingly, we sought to optimize a protocol for the isolation of both soluble and aggregated amyloid proteins from human brain tissues for quantitative proteomic analysis. Previous attempts have studied amyloid proteins using detergents/detergent-free buffers together with sequential extraction and quantification by ELISA, immunoblotting, or immunocytochemistry [9–11], but these approaches are unable to determine the aggregation state of the amyloids. More rarely, attempts have been made to determine the complete composition of amyloid plaques and to understand how the constituent proteins contribute to plaque formation, but this method disregards any involvement of non-amyloid proteins in the aggregation process. Our strategy was therefore to use mass spectrometry-based proteome-wide profiling of both the soluble and aggregated fractions of human brain tissues affected by dementia, thereby shedding new light on potential mechanisms of protein aggregation and plaque formation in common neurodegenerative diseases.

The human brain proteome is extremely complex and requires robust techniques to enable accurate protein identification and high proteome coverage. Ultracentrifugation (UC) is a very effective method of sample sedimentation that facilitates the recovery of aggregated proteins and small particles including ribosomes, extracellular vesicles, viruses and rare protein variants from heterogeneous biological materials [12, 13]. After sample processing, electrostatic repulsion-hydrophilic interaction chromatography (ERLIC) can be used to fractionate the sample and facilitate separation of peptide isoforms with different charges such as deamidated peptides by mass spectrometry. The objective of our study was to use this combined UC-ERLIC approach to conduct a comprehensive proteomic analysis of human brain tissue samples and identify dementia-associated changes in amyloid protein composition, relative abundance, and extent of deamidation. Our data reveal that amyloid plaque formation is associated with protein deamidation in human brain tissue. We identified specific proteins that were deamidated only in the insoluble aggregates, and structural modeling of these modified proteins predicted changes in charge state and ion binding potential that would be expected to promote aggregation. This study reports for the first time that human brain proteins exhibit extensive deamidation in dementia-associated amyloidosis, and suggests that deamidation promotes protein aggregation and amyloid plaque formation in human neurodegenerative disease.

## Results

### Optimizing brain protein extraction to prevent sample aggregation *ex vivo*

Amyloidosis is the process by which malformed proteins aggregate and form insoluble plaques in body tissues, eventually leading to impaired organ function. While amyloidosis has been implicated in the pathology of more than 20 major human diseases, including several neurodegenerative disorders, the variable composition and solubility of amyloid deposits have so far prevented detailed analysis of their role in human cognitive impairment. We therefore sought to develop robust methods to facilitate proteomic analyses of human brain tissues and identify disease-associated changes in the brain proteome that might shed new light on the process of amyloidosis in human neurodegeneration. In order to accurately identify brain proteins that undergo aggregation in vivo, it is essential to prevent artifactual protein aggregation during sample processing in vitro. Since protein aggregation and structural modifications are pH-sensitive events that can be triggered by inappropriate buffer use, we first tested the efficiency of protein extraction from brain tissue samples using a range of mildly acidic and alkaline buffers (AAB, ABB and TEAB; see methods) at various different temperatures (10, 22 and 37 °C) for variable duration (1, 3 or 7 days). The extracted soluble and aggregated proteins in all preparations were measured and analyzed by LC-MS/MS. We observed that optimal protein extraction was achieved using mild detergent (2 % SDS) together with a weak acid pH buffer (AAB, pH 6.0), which generated a high total protein yield, restricted sample aggregation during processing, and protected against structural changes in vitro. These data demonstrated that via extensive protocol optimization, it is possible to conduct robust proteomic analyses of primary human brain tissues, thereby facilitating the identification of specific amyloids, changes in plaque composition, and structural modifications of brain proteins that might promote amyloidosis and/or disease progression in human neurodegenerative disorders.

### Enrichment of human brain amyloidal proteins

**Fig. 1:**
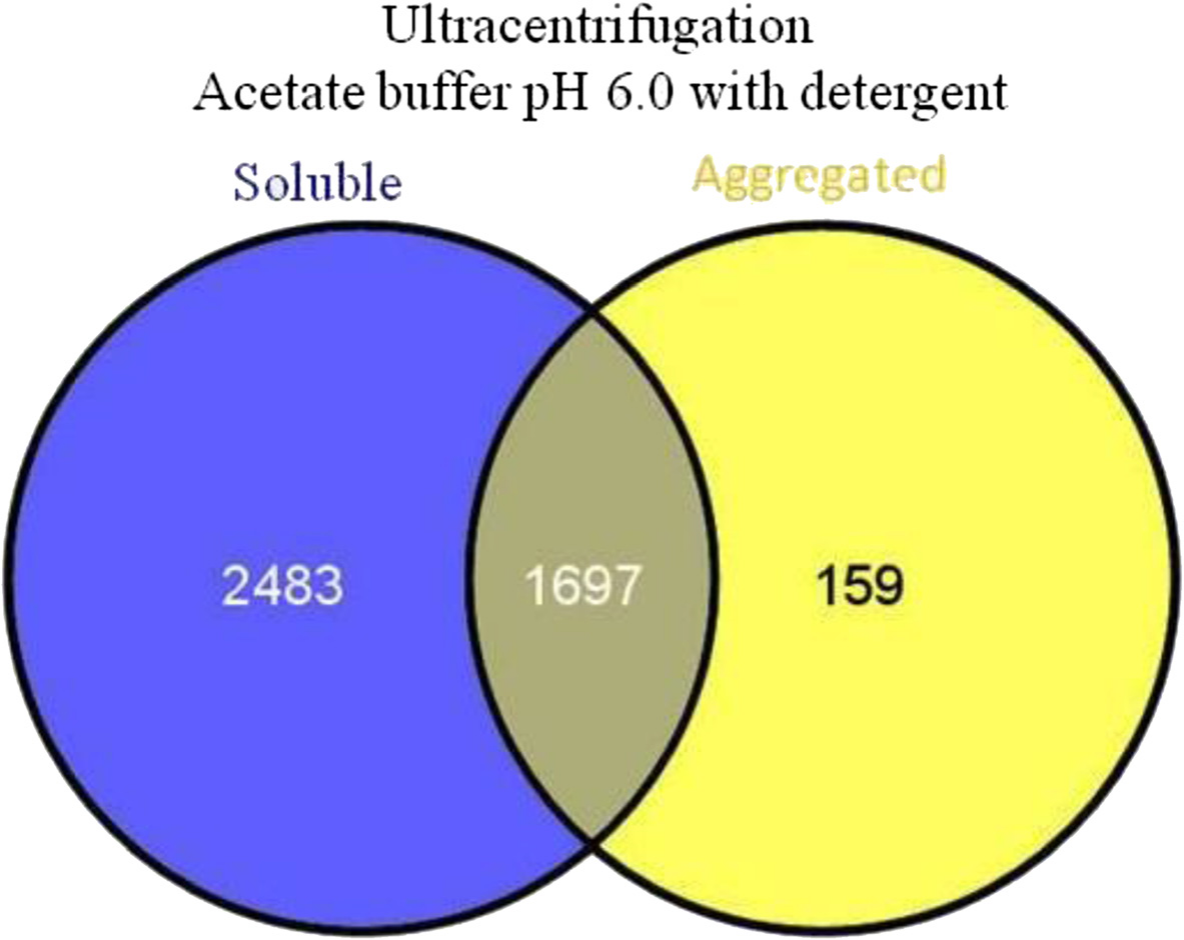
Venn diagram showing overlap of the brain proteins identified in the soluble and aggregated fractions. Proteins were extracted using acetate buffer pH 6.0 with detergent

**Fig. 2:**
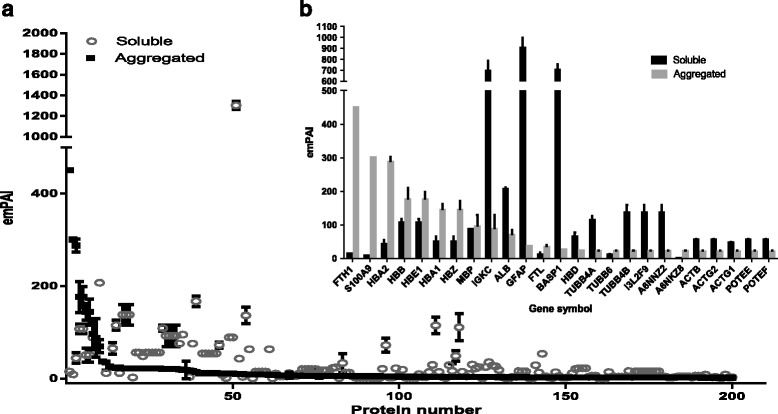
Protein abundance in the aggregated fraction of the human brain proteome as extracted using detergent/acetate buffer together with ultracentrifugation/ERLIC fractionation (**a**). Embedded panel details the most abundant proteins detected in the aggregated fraction (**b**)

**Fig. 3:**
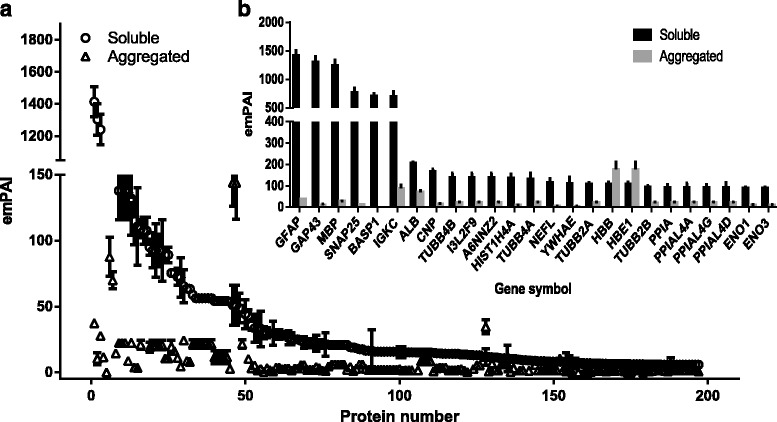
Protein abundance in the soluble fraction of the human brain proteome as extracted using detergent/acetate buffer together with ultracentrifugation/ERLIC fractionation (**a**). Embedded panel details the most abundant proteins detected in the soluble fraction (**b**)

**Fig. 4:**
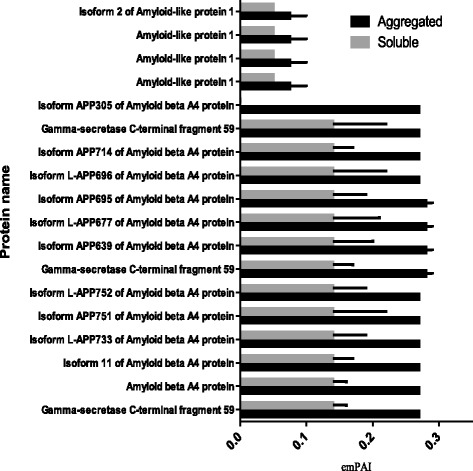
Relative abundance of different forms of APP in the soluble and aggregated protein fractions of the human brain proteome

Protein aggregation into amyloid plaques is a pathologic hallmark of multiple neurodegenerative diseases, but therapies directed against amyloid proteins have shown little clinical benefit in human patients so far, suggesting a critical role for non-amyloid proteins in the formation of plaques. Having optimized our protocol for extraction of both soluble and aggregated proteins from post-mortem samples of human brain, we next conducted a proteome-wide analysis of amyloid structure, relative abundance and plaque composition in human brain tissues affected by dementia. To do this, we extracted both soluble and aggregated amyloidal proteins from brain using our UC-based method, subjected the aggregated proteins to further extraction and solubilization in formic acid (FA), and separated the deamidated peptides by ERLIC fractionation and structural analysis by LC-MS/MS. Using this approach, we identified a total of 5225 ± 248 brain tissue proteins, of which 4339 ± 104 individual proteins remained after application of a stringent false-discovery rate (FDR; cutoff value <1.0 %) and the characterized proteins have been identified with at least 2 unique peptides. Overlap analysis revealed that while 1697 proteins were present in both the soluble and aggregated fractions, 159 individual brain proteins were specific to the aggregated (pellet) fraction only (Fig. 1). We observed significant enrichment of S100A9, ferritin, mitochondrial creatine kinase (U-type), and hemoglobin subunits (α, β, ε and ζ) within the protein aggregate (Fig. 2a, b), as well as increased abundance of collagen, palmitoyl-protein thioesterase 1, laminin, coronin-1A, S100-B, S100-A8, Syntaxin-binding protein 2, cathepsin G, grancalcin, and syntaxin-binding protein 2 (Additional file 1: Table S1A–D). While protein S100A9 was present in both sample types, the levels detected in the pellet fraction (protein score 5083.50 ± 172.50, emPAI 300.74 ± 0.00) were far higher than those detected in the soluble fraction (1315.50 ± 119.50; 8.81 ± 0.00), suggesting substantial enrichment of S100A9 in brain amyloid aggregates (Fig. 2, Additional file 2: Table S2 and Additional file 1: Table S1C). Similarly, the iron-storing protein ferritin was also enriched in the pelleted protein aggregate (emPAI 450.45 ± 0.00) relative to the levels detected in the soluble fraction (15.20 ± 0.00; Fig. 2b). Also enriched in the pellet fraction were the hemoglobin subunits α, β, ε and ζ, consistent with reports that elevated hemoglobin levels are associated with increased risk of neurodegenerative disease and rapid cognitive decline (Additional file 2: Table S2 and Additional file 1: Table S1D). Conversely, a distinct subset of brain proteins was more abundant in the soluble fraction than in the pellet, including neuromodulin, glial fibrillary acidic protein, myelin basic protein isoform 4, synaptosomal associated protein 25, brain acid soluble protein 1, and components of β-tubulin (Fig. 3a and b). After further separation of the soluble fraction by UC-ERLIC, we were also able to detect several different isoforms of APBB2 and amyloid-like protein APLP2, as well as APBB1, APBB1IP and SAA4. These data indicated that multiple amyloid-like protein isoforms displayed only intermediate levels of aggregation and were therefore largely absent from the pellet fraction (Fig. 4).

### Brain protein deamidation in dementia

Accumulation of structural modifications that impair protein function is a common feature of multiple age-related pathologies including major neurodegenerative diseases. Alterations including deamidation, racemerization and glycation have all been reported to disrupt normal protein function, and deamidation in particular is known to induce major perturbations in protein structure, folding/topology and overall stability. In the current study, we observed extensive deamidation of brain proteins in the pelleted insoluble aggregate, including S100A9, ferritin, hemoglobin, creatine kinase (U-type), S100-B, collagen α-2(IV) chain, collagen α-2(I) chain, laminin subunit β-2, dystonin (isoform 3), and serine/threonine-protein kinase (isoform 2) (Additional file 2: Table S3 and Additional file 1: Table S1E). We also detected deamidation of proteins coronin-1A and syntaxin-binding protein 2, which have previously been implicated in neurodegeneration of the hippocampus. Importantly, the presence of deamidated proteins was not restricted to the aggregated pellet, since the soluble brain proteome also exhibited extensive deamidation of creatine kinase B. In parallel, we observed that mitochondrial creatine kinase was highly enriched in the pellet and deamidated exclusively within the aggregate, suggesting that organelle dysfunction and compromised energy metabolism may be a feature of brain plaque formation and neurodegeneration.

**Fig. 5:**
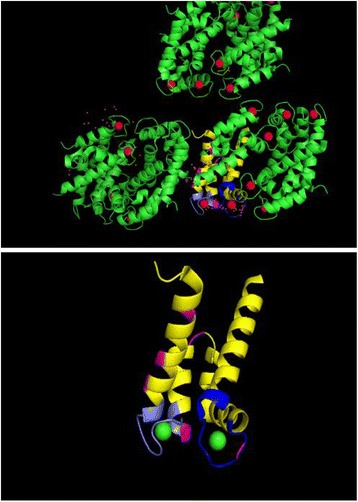
Structural model of Protein S100A9 (RCSB Protein Data Bank accession code: 1XK4) showing deamidation sites. EF hand calcium binding motifs are shown in yellow and deamidation sites (area of modification) are highlighted in magenta and blue. Helices are shown in green while Ca ions are shown as red spheres. Lower panel shows only EF hands and area of modification with Ca ions in green

Many specific deamidation sites were exclusively identified in the aggregated proteins, including modifications of S100A9, ferritin, hemoglobin subunits, S100-A8, S100-B, collagens, mitochondrial creatine kinase (U-type), β-tubulin and laminin. The deamidation sites of protein S100A9 that were commonly identified in Mascot and Maxqunat were listed in Table 1. These specific structural modifications may have functional consequences that influence the progression of neurodegenerative diseases. For example, the inflammation-associated calcium binding protein S100A9 incorporates two EF-hand motifs that exhibit differential affinity for Ca^++^ binding, and deamidation of both motifs was detected exclusively in the aggregated fraction (Fig. 5). In contrast, only low levels of S100A9 were detected in the soluble fraction and the protein was not deamidated in this sample. These data suggest that deamidation and aggregation of brain proteins that regulate inflammatory processes may contribute to the pathology of human neurodegeneration. While these data suggested that protein deamidation and aggregation are key features of brain plaque formation, we also detected the restorative enzyme PIMT (ProteinL-isoaspartate O-methyltransferase) within the pellet aggregate, perhaps indicating a failed response to repair disrupted protein functions. In vitro incubation of protein samples at various different temperatures (10, 22 and 37 °C) for variable duration (1, 3 or 7 days) in different buffers like AAB (pH 6), ABB (pH 8) and TEAB (pH 8.5) revealed precipitation of protein except acetate buffer (pH 6). As alkaline condition is known to induce protein deamidation, this observation may suggest that deamidation causes aggregation of proteins.

**Table 1:** S100A9 peptide sequences and deamidation sites commonly identified in Mascot and Maxqunat that were highly enriched among the aggregated brain proteins

Protein accession number	Protein description	Peptide sequence and site of modification (Mascot)	Peptide sequence and site of modification (Maxquant)
P06702	Protein S100-A9	K.LGHPDTLN#QGEFK.E	KLGHPDTLN#QGEFKE
P06702	Protein S100-A9	K.LGHPDTLNQ#GEFKELVR.K	KLGHPDTLNQ#GEFKE
P06702	Protein S100-A9	K.VIEHIMEDLDTN#ADK.Q	VIEHIMEDLDTN#ADK
P06702	Protein S100-A9	R.N#IETIINTFHQYSVK.L	NIETIINTFHQ#YSVK
P06702	Protein S100-A9	R.NIETIIN#TFHQYSVK.L	NIETIIN#TFHQYSVK
P06702	Protein S100-A9	R.NIETIINTFHQ#YSVK.L	NIETIINTFHQ#YSVK
P06702	Protein S100-A9	R.N#IETIINTFHQYSVK.L	N#IETIINTFHQYSVK

## Discussion

Aggregation of brain proteins is thought to be a key feature of human neurodegenerative diseases. While some studies have successfully linked protein plaque formation with disease severity [14, 15], other investigators have failed to confirm this correlation [16, 17], perhaps because amyloid deposition represents an early event that precedes clinical symptoms [18], or possibly due to key biochemical changes occurring outside the plaques. Indeed, soluble amyloid oligomers can form even in the brains of cognitively normal individuals where they are known to be highly neurotoxic [19], and there is extensive evidence to suggest that non-plaque, water-soluble amyloids contribute to the pathology of neurodegenerative pathology [20]. Previous studies have reported that levels of soluble amyloid detected by Western blotting are significantly correlated with frequency of neuritic plaques and neurofibrillary tangles in AD [21]. Similarly, Naslund et al., observed no link between plaque number and cognitive impairment in their study of patients with dementia, but the quantity of the insoluble amyloid plaque did correlate with declining brain function [22]. While previous studies have sought to quantify specific isoforms of soluble amyloids using ELISA analysis of mouse and human brain tissues [9, 11], the potential role played by these proteins in the neurodegenerative process has remain unclear (reviewed in [23]). By applying an unbiased discovery-based proteomics approach in the current study, we now provide the first comprehensive profile of both the soluble and aggregated fractions of the human brain proteome in dementia, and we present evidence that deamidation leading to impaired protein function may play a significant role in disease pathology.

Deamidation adds a negative charge at sites of modification and thereby promotes protein aggregation, which is a pathological hallmark of several age-related disorders and common neurodegenerative diseases [24–29]. Accordingly, protein deamidation has been strongly implicated in the pathogenesis of dementia syndromes [30, 31], and in the current study we observed that deamidation was a common feature of aggregated brain proteins including ferritin, S100 family members, hemoglobin subunits, collagens, mitochondrial creatine kinase (U-type), β-tubulin and laminin. Collagen type XXV is a key component of amyloid-containing plaques in the brains of patients with neurodegenerative diseases [32], and here we observed deamidation of collagen chains α-2(IV) and α-2(I) which might be predicted to increase aggregation. Furthermore, we detected significant enrichment of ferritin in aggregated proteins from brain tissues affected by dementia, consistent with previous reports that increased ferritin iron is associated with neurodegenerative pathology [33]. It is possible therefore that deamidation and aggregation of ferritin leads to increases in iron content in the brain during neurodegeneration. These data suggest that protein functional impairment due to deamidation of key residues may have a major role to play in shaping neurodegenerative pathology. Indeed, a previous study has reported that deamidation contributes to insolubilization and aggregation of lens protein crystallins during cataract formation [34].

It has been reported that AD patients exhibit decreased expression of creatine kinase (U-type) in the brain, suggesting altered energy metabolism in affected tissues [35]. In the current study we instead observed substantial enrichment of this enzyme in the pellet fraction, although the protein also exhibited marked deamidation in the aggregate, suggesting that normal metabolic function may be disrupted even when creatine kinase levels are normal. Formation of creatine kinase crystalline inclusions has been identified in the mitochondria of cells subjected to ischemia [36]. The presence of these inclusion bodies is associated with oxidative adaptation to restriction of energy supply, and is a clinical hallmark of mitochondrial cytopathy in multiple neuropathologies including dementia [37, 38]. Deamidation of creatine kinase B is also associated with loss of enzyme activity in PIMT knockout mice [38, 39], which exhibit severe neuropathology with rapid onset after birth. In the current study, we identified high levels of PIMT in the insoluble protein pellet fraction, suggesting that aggregation of this enzyme disrupts a key mechanism of protection against further protein deamidation (as has already been reported in aging brain tissues in both human and animal models [40, 41]). However, it is important to note that among the identified proteins already implicated in dementia pathology, many were enriched in the soluble fraction rather than the aggregated pellet (including GFAP, GAP43, MBP, SNAP25, BASP1, IGKC, TUBB4B, TBB8L, TUBB4A [42–44]), suggesting that full elucidation of disease pathogenesis will require a better understanding of changes in brain protein structure and function outside of the plaque.

In our study of human brain tissue affected by dementia, we observed that the pelleted aggregate was significantly enriched in the demidated protein S100A9, consistent with reports that knockdown of S100A9 can restore cognitive function and reduce amyloid plaque burden in the Tg2576 mouse model of AD [45]. In line with these findings, it has previously been proposed that S100A9 promotes the formation of β-amyloid aggregates [19], although the molecular mechanism by which this proteinopathy might be initiated remains unknown. By mapping the deamidation sites identified onto the 3D structure of S100A9, we observed that both the low- and high-affinity calcium binding motifs that characterize this protein [46] were deamidated only in the aggregated fraction. Since deamidation is thought to introduce negative charge to the modified sites of the affected protein [30, 31], deamidation of the S100A9 calcium binding sites could therefore be predicted to promote protein aggregation and amyloid formation in the brain. S100A9 composed of two EF-hand motifs: the N-terminal motif comprising helices I and II, and, separated by a flexible linker, the C-terminal motif with helices III and IV, thus providing two Ca^2+^-binding sites. The details of the structure have been described by Itou et al. [47] and Korndörfer et al. [46]. Deamidation is a spontaneous process and validation of deamidation at specific sites is very much challenging due to lack of commercial antibodies. However, when proteins were incubated in vitro at various different temperatures (10, 22 and 37 °C) for variable duration (1, 3 or 7 days) in different buffers like AAB (pH6), ABB (pH8) and TEAB (pH8.5), we found precipitation of proteins in alkaline buffers, but not in acetate buffer at (pH 6.0). As protein is prone to deamidation at pH > 7, this support that protein deamidation causes aggregation of proteins. Secretion of S100A9 during inflammation has previously been reported to enhance the formation of amyloid plaques [19], and our data now suggest that deamidation of this protein could be a key event in amyloidosis in the human brain. Novel therapies that prevent deamidation of brain proteins such as S100A9 could therefore represent an effective approach to the treatment of human neurodegenerative diseases.

## Conclusions

Localized deposition of amyloid protein in the brain is an invariant and defining feature of several neurodegenerative diseases. Previous attempts to characterize human amyloidal plaque composition have been infrequent and met with only limited success. We therefore developed a novel approach (UC-ERLIC) to the isolation of both soluble and aggregated amyloidal proteins from human brain tissues and subjected these to comprehensive proteomic profiling. This study demonstrated significant enrichment and deamidation of S100A9, ferritin, hemoglobin subunits, and myelin basic protein among the aggregated brain proteins. Protein S100A9 was highly abundant in the amyloidal plaque samples, and structural analysis indicated that both the low- and high-affinity calcium binding motifs of S100A9 were deamidated exclusively in the aggregated fraction, suggesting altered charge state and function of this protein in brain tissues affected by dementia. Together, these data reveal for the first time that deamidation of S100A9 may represent a key mechanism that triggers protein aggregation and amyloid formation in the human brain.

## Methods

### Extraction of soluble and aggregated proteins from human brain tissue

Sequencing-grade modified trypsin (V5111) was from Promega (Madison, WI, USA). Protease inhibitor cocktail tablets (cOmplete) were from Roche (Basel, Switzerland). All other chemicals and reagents were from Sigma–Aldrich (St. Louis, MO, USA) unless otherwise stated. Post-mortem brain tissue samples of a 69-year-old male diagnosed with subarachnoid hemorrhage and dementia issues were obtained from the brain bank of the Choju Medical Institute of the Fukushimura hospital (Toyohashi, Aichi, Japan), and the protocols utilized were approved by the local ethics committee of the Fukushimura hospital. The scientific use of human material was conducted in accordance with the Declaration of Helsinki, and informed consent was obtained from the guardians of the patients [48]. All procedures were approved and performed in accordance with the ethical guidelines of the Nanyang Technological University ethics board. The tissue was first dissected, the large blood vessels removed, and the remainder cut into small pieces and homogenized. In initial experiments, we used 12.5 ml ice-cold homogenization buffer (2 % w/v SDS, 20 mM Tris–HCl, pH 7.4) supplemented with Roche cOmplete protease inhibitor cocktail. In later experiments, protein sample processing was performed under mildly acidic conditions (2 % SDS, 50 mM ammonium acetate buffer, pH 6.0) in order to minimize deamidation artifacts and eliminate false positive identification [49]. After tissue homogenization, cell debris was removed by centrifugation at 3000 × g, 10 °C for 10 min, then the supernatant was ultracentrifuged at 112000 × g, 10 °C for 1 h. The resultant supernatant was collected and subjected to two further rounds of ultracentrifugation at 112000 × g, 10 °C for 1 h. The amyloid-enriched pellets generated by these two ultracentrifugation steps were combined and re-suspended in 12.5 ml mild acid buffer (2 % SDS, 50 mM ammonium acetate buffer (AAB), pH 6.0) and then mixed and ultracentrifuged at 112000 × g, 10 °C for 1 h. The supernatants generated by each of the ultracentrifugation steps were pooled for later analysis. The pellet containing the insoluble amyloidal plaque was re-suspended in 1 ml formic acid (FA; 70 %), then vortexed for 2 min, and centrifuged for 15 min at 18000 × g, 4 °C. To ensure complete dissolution, the final sample pellet was re-suspended in 1 ml FA (99 %), vortexed for 2 min, and centrifuged at 18,000 × g, 4 °C for 10 min. To support protein deamidation causes protein aggregation, in vitro extracted protein were incubated in buffers like ammonium acetate buffer (AAB, 50 mM, pH 6.0), triethylammonium bicarbonate (TEAB, 50 mM, pH8.5), ammonium bicarbonate buffer (ABB, 50 mM, pH 8.0), at different temperatures (10, RT (22 °C) and 37 °C) and different durations (day 1, day 3 and day 7).

### Sample preparation and LC-MS/MS analysis

Proteins were reduced with dithiothreitol (10 mM), alkylated using iodoacetamide (55 mM) and then subjected to overnight digestion in sequencing-grade modified trypsin at 37 °C. Tryptic peptides were fractionated by ERLIC separation on an amine-column using a Shimadzu Prominence UFLC system. Mobile phase A (85 % acetonitrile, 0.1 % acetic acid) and phase B (10 % acetonitrile, 0.1 % FA) were used to establish a 60 min gradient. The eluted fractions were collected at 1 min intervals, pooled into 26 separate fractions for each sample, then dried and reconstituted in 0.1 % FA.

Peptides were separated and analyzed on a Dionex Ultimate 3000 RSLC nanoLC system coupled to a Q-Exactive apparatus (Thermo Fisher, MA). Approximately 5 μl sample was injected into an acclaim peptide trap column via the autosampler of the Dionex RSLC nanoLC system. The flow rate was set at 300 nl/min. Mobile phase A (0.1 % FA in 5 % acetonitrile) and mobile phase B (0.1 % FA in acetonitrile) were used to establish a 60 min gradient. Peptides were then analyzed on a Dionex EASY-spray column (PepMap® C18, 3um, 100A) using an EASY nanospray source at an electrospray potential of 1.5 kV. A full MS scan (350–1600 m/z range) was acquired at a resolution of 70,000 at m/z 200, with a maximum ion accumulation time of 100 ms. Dynamic exclusion was set to 30 s. Resolution for MS/MS spectra was set to 35,000 at m/z 200. The AGC setting was 1E6 for the full MS scan and 2E5 for the MS2 scan. The 10 most intense ions above a 1000 count threshold were selected for HCD fragmentation, with a maximum ion accumulation time of 120 ms. An isolation width of 2 Da was used for the MS2 scan. Single and unassigned charged ions were excluded from MS/MS. For HCD, normalized collision energy was set to 28. The underfill ratio was defined as 0.1 %.

### Data analysis

Raw data files were converted into the mascot generic file format using Proteome Discoverer version 1.4 (Thermo Electron, Bremen, Germany) with the MS2 spectrum processor for de-isotoping the MS/MS spectra. The concatenated target-decoy UniProt human database (sequence 88 473, downloaded on 29 November 2013) was used for data searches. The database search was performed using an in-house Mascot server (version 2.4.1, Matrix Science, Boston, MA) with MS tolerance of 5.1 ppm and MS/MS tolerance of 0.02 Da. Two missed trypsin cleavage sites per peptide were tolerated. Carbamidomethylation (C) was set as a fixed modification, while oxidation (M) and deamidation (N and Q) were variable modifications. Annotated MS/MS spectra of Mascot-detected deamidated peptides were exported and showed in Supplementary data (Annotated MS_MS spectra _Mascot). Data were then searched again using the same criteria in MaxQuant (version 1.5.2.8) [50] to ensure maximum confidence protein identification, quantification and assessment of deamidation status. In Mascot, label-free quantitation (LFQ) of proteins uses emPAI [51], which is based on spectral counting, whereas LFQ in Maxquant is based on the ion intensities of the extracted ion chromatogram [52]. For the first search, peptide mass tolerances were 20 ppm and 4.5 ppm respectively, whereas for MS/MS the threshold was 20 ppm with FTMS de-isotoping enabled. The search was performed in revert decoy mode with PSM FDR, protein FDR and site decoy fraction set at 0.01. Annotated MS/MS spectra of Andromeda-detected deamidated peptides were exported and showed in Supplementary data (Annotated MS_MS spectra_Maxqunat). Proteins were considered for further analysis only when identified by both software packages as displaying differential abundance in the soluble and aggregated fractions, and featured deamidation sites that were consistently identified by both search engines.

### Ethics approval and consent to participate

Post-mortem brain tissue samples were obtained from the brain bank of the Choju Medical Institute of the Fukushimura hospital (Toyohashi, Aichi, Japan), and the protocols utilized were approved by the local ethics committee of the Fukushimura hospital. The scientific use of human material was conducted in accordance with the Declaration of Helsinki, and informed consent was obtained from the guardians of the patients. All procedures were approved and performed in accordance with the ethical guidelines of the Nanyang Technological University ethics board.

### Consent for publication

Not Applicable.

### Availability of data and material

Raw mass spectrometry data and quantification results from MaxQuant (including protein and peptide identification) have been deposited to the ProteomeXchange Consortium [53] via the PRIDE partner repository with the dataset identifier PXD002516.

## Additional files

Additional file 1: Table S1A. Total Proteins (including contaminants and decoy hits) identified in soluble and pellet (aggregated) fraction by Maxquant. **Table S1B**. Human proteins identified in soluble and pellet (aggregated) fraction by Maxquant. **Table S1C**. Human proteins enriched in aggregated fraction as identified in both soluble and pellet (aggregated) fractions by Maxquant. **Table S1D**. Human proteins enriched in aggregated fraction as identified mainly in pellet (aggregated) fraction by Maxquant. **Table S1E**. Unique deamidated peptides identified in pellet fraction and sites of modifications. Annotated MS_MS spectra_in Maxquant Annotated MS_MS spectra in Mascot Peptide View. (XLSX 6872 kb) Additional file 2: Table S2. Proteins identified in soluble and pellet (aggregated) fraction by Mascot along with their emPAI. **Table S3**. Unique deamidated peptides identified in pellet fraction and sites of modifications. (XLSX 740 kb)

## Abbreviations

UC-ERLIC: ultracentrifugation-electrostatic repulsion hydrophilic interaction chromatography
AD: alzheimer’s disease
PD: parkinson’s disease
HD: huntington’s disease
ERLIC: electrostatic repulsion-hydrophilic interaction chromatography
LC-MS/MS: liquid chromatography-tandem mass spectrometry
AAB: ammonium Acetate buffer
ABB: ammonium Bicarbonate Buffer
TEAB: triethylammonium bicarbonate buffer
SDS: sodium dodecyl sulfate
FA: formic Acid
FDR: false discovery rate
PIMT: Protein L-isoaspartate O-methyltransferase
LFQ: label free quantitation
PSM FDR: peptide-to-spectrum match false discovery rate
